# Cotton Instead of Lint for Surgical Uses

**Published:** 1834

**Authors:** 


5. *Cotton instead of lint for surgical uses.*—It has been objected to cotton as a surgical dressing, that it is too irritating; but a late French writer (*Boston Journal*) speaks on this subject in the following language:

"On more than one occasion the attempt has been made to replace lint in the dressing of wounds, by a substance more easy of access of a less price, and less susceptible of acquiring injuries. We have already in this journal directed the attention of surgeons and of the managers of hospitals to the charpie of hemp, the use of which ought already to have been more general in the army and in temporary hospitals. We shall now attempt to describe the properties of cotton as a dressing. Cotton, which like lint is a vegetable substance, has the advantage of being lighter, and is so abundant as to be sold at a very low price. It is easily applied, without previous preparation, in thin and equal flakes. By doubling the usual flake, we obtain at pleasure two soft surfaces, like the English lint, and which may be applied to the same uses as this tissue. Once in place, the cotton is not subject to disturbance, and remains attached on wounds and ulcers, even of the face, without its being necessary to sustain it by means of bandages or plasters. Lastly, it has more elasticity than lint, and preserves this precious property in a variety of circumstances, and even when it becomes damp. It is indeed objected to it, that like wool, it is full of points and motes, which render it irritant and injurious; but by applying a few filaments of it under the eyelids, on a fresh wound, a burn, or blister, it will easily be seen what becomes of these asperities. The opposite objection has also been made, namely, that the cotton did not excite granulations, but produced an imperfect pus, by furnishing an insufficient stimulus. This objection is as ill founded as the last; and to be convinced of it, we need only try a few experiments, which are equally free from expense and from danger.

"In regard to economy, a simple comparison of the relative prices of lint and of cotton will make this truth evident. Fine lint for equal weight costs more than cotton of prime quality; but the difference in volume is such, in consequence of the specific levity of the latter, that a quarter of a pound of the one equals a pound of the other, and that with equal weights the cotton will cover a surface four or five times more considerable than the other. Still more, cotton being applicable to a variety of purposes, and capable of being cleansed perfectly by chemical processes, that which has already served for dressings will command a considerable proportion of its former price, which cannot be said of lint."

